# Paraumbilical hernia repair during cesarean delivery

**DOI:** 10.4103/0256-4947.51798

**Published:** 2009

**Authors:** Wagih M. Ghnnam, Adel S. Helal, Muhammad Fawzy, Ahmed Ragab, Hend Shalaby, Ehsan Elrefaay

**Affiliations:** a Department of General Surgery, Mansoura Faculty of Medicine Mansoura University, Mansoura, Dakahlia, Egypt; b Department of Obstetrics and Gynecology, Mansoura Faculty of Medicine Mansoura University, Mansoura, Dakahlia, Egypt

## Abstract

**BACKGROUND AND OBJECTIVES:**

Pregnant women with paraumbilical hernia usually postpone hernia repair until after delivery, but some patients request that it be done during cesarean delivery. Therefore, we evaluated the outcome of combined cesarean delivery and paraumbilical hernia repair in a prospective study at a tertiary referral university hospital.

**PATIENTS AND METHODS:**

In a prospective study, we compared the outcome of 48 patients undergoing cesarean delivery combined with paraumbilical hernia repair versus 100 low-risk patients undergoing cesarean delivery alone. The main outcome measures were operation time, blood loss, severity of pain, peripartum complications, hospital stay, hernia recurrence, and patient satisfaction.

**RESULTS:**

The combined procedure took significantly longer than cesarean delivery alone (75.2 minutes versus 60.5 minutes, *P*<.001)). There were no major complications. Wound infection occurred in 6 patients (4.1%). Hospital stay did not differ significantly from those of controls. Pain at the hernia site repair occurred in two patients, and one hernia recurred in the hernia repair group during a mean follow-up period of 22 months (range, 6-36 months). All hernia patients reported that they preferred the combined operation.

**CONCLUSIONS:**

Combined cesarean delivery and paraumbilical hernia repair had the advantage of a single incision, single anesthesia, and a single hospital stay while avoiding re-hospitalization for a separate hernia repair. Our results indicate that the combination approach is safe, effective, and well accepted.

Umbilical hernias account for 6% of all abdominal wall hernias in adults.^1^ There is no consensus on the best technique for the repair of umbilical hernia and thus various surgical techniques such as primary suture, Mayo repair, mesh repair, and laparoscopic surgery have been used for the treatment of this surgical condition.^2^ Cesarean delivery has been practiced over the centuries, but only relatively recently has it became such a safe procedure that women are requesting that it be used to deliver their baby in the absence of any other indication.^3^ The combination of umbilical hernia repair with gynecologic surgery or cesarean delivery is virtually undocumented except for a case report from 1987^4^ and another report of 8 patients having inguinal and umbilical hernia repaired during cesarean delivery.^5^ However, the combination of paraumbilical hernia repair with abdominal surgery remains unstudied except in conjunction with cholecystectomy.^6^,^7^ The practice is uncommon, probably because most obstetricians believe that any additional risk from combined surgery is unwarranted since repair of paraumbilical hernia is rarely urgent. We have offered a hernia repair in conjunction with cesarean delivery since 2004 to patients with a paraumbilical hernia during pregnancy (if a cesarean delivery were planned). The aim of this study was to compare the outcome in this group with that of a low-risk control group undergoing cesarean delivery alone at the same institution.

## PATIENTS AND METHODS

In a prospective study, we compared the outcome of 48 pregnant women who underwent a paraumbilical hernia repair in combination with a scheduled elective cesarean delivery between January 2004 to January 2006 with that of a control group of 100 pregnant women who underwent caesarean delivery alone during the same period. Outcome measures assessed included operative time, postoperative complications, and length of hospital stay. The control group were free of medical problems as diabetes, heart disease, and anemia or other conditions that might place them at greater risk of complications. The 48 women provided informed consent to undergo cesarean delivery combined with primary hernia repair. Cesarean delivery was conducted as follows: the skin was disinfected with bovidine iodine; a Pfannenstiel skin incision was made in the lower crease; the fetus was delivered; the uterine wound was closed, all the while maintaining good hemostasis. All patients received antibiotic prophylaxis in the form of intravenous cefotaxime sodium 1 gram after placental extraction. In 36 patients who had a defect less than 3 centimeters we performed umbilical hernia repair by means of a primary suture from the inside. In 12 patients with a defect more than 3 centimeters, repair was performed by inside mesh hernioplasty fixed to the peritoneum and sheath by non-absorpable polypropylene suture. Good peritoneal toilet and closure of the cesarean wound was done as usual. Postoperative pain was evaluated by the average consumption of analgesic ampules given on patient demand during the first week. All patients received a routine intramuscular dose of 75-mg dose of diclophenac sodium (Voltaren IM; Novartis, Basel, Switzerland) twice on the day of the operation. The same analgesic was given as needed later. Total analgesic consumption was recorded during the first postoperative week. Wound infections, separations, seromas and hematomas were treated with local care and/or antibiotics, on an outpatient or in-patient basis as the situation required. Wound infection was diagnosed if the wound drained purulent material or the incision required opening and showed two or more of the classical signs of infection, such as erythema, tenderness, induration, and fever. Wound seroma or hematoma was diagnosed in the presence of a serous fluid collection or subcutaneous blood without signs of infection. Wound disruption was defined as spontaneous or iatrogenic separation of the wound edges more than 1 cm. The patients were followed-up postoperatively daily until discharge from the hospital, then every 3 days as an outpatient for the first 2 weeks, then 1 month later, and finally every 6 month. Descriptive statistics are shown as the mean±standard deviation. Statistical analyses were done with the t test, MannnWhitney U test, and KruskalnWallis test as appropriate.

## RESULTS

There were no significant differences in age, parity or gravidity between the patients that had cesarean delivery plus hernia repair (group I) and those who had cesarean delivery alone (group II) (Table 1). Repeated cesarean delivery was the most common indication for cesarean delivery in both groups (other indications are listed in Table 2). Mean operative time was significantly longer (*P*<.001) in group I compared with group II, but the mean length of hospital stay was similar in the two groups (*P*=.21) (Table 2). No significant difference was found between pren and postoperative mean hemoglobin levels (Table 3). The patients in group I reported more pain; the need for analgesics was significantly less in group II (*P*=.027). The rate of early and late postoperative complications such as seroma and/or hematoma, wound infection, and wound disruption was similar in the two groups (Table 3). There was one recurrence (2.8%) in the suture repair subgroup, whereas no recurrence was detected in the mesh hernioplasty subgroup. The mean follownup time was 22 months (range, 6 to 36 months). Caesarean delivery suture healing was delayed, with wound infection in 6 patients (2 in group I and 4 in group II). The patients expressed subjective satisfaction and preferred the combined procedure. No postoperative mortality was observed. The recurrence rate in patients in the suturenreceiving subgroup was statistically significant when compared with patients in the mesh-receiving subgroup (*P*<.03).

**Table 1:** Age, parity and gravidity in the study population (group I: combined hernia and cesarean delivery; group II: cesarean delivery alone).

	Group I (n=48)	Group II (n=100)	F ratio	*P* value
Age (y)	30.6±2.6	28.6± 2.6	1.057	.8
Parity	2.1±1.1	1.5±1.0	1.125	.61
Gravidity	3.4±1.1	2.7±1.0	1.159	.53

Values are mean±SD. CD: cesarean delivery

**Table 2:** Indications for cesarean delivery, operative time, hospitalization time and pre- and postoperative mean hemoglobin in the study population (group I: combined hernia and cesarean delivery; group II: cesarean delivery alone).

	Group I (n=48)	Group II (n=100)	*P* value
Indications for CD			
Primary cesarean delivery			
CPD	1 (2.8%)	2 (2.0%)	.7
Failure to progress	9 (18.8%)	18 (18.0%)	.9
Malpresentation	4 (8.3%)	9 (9.0%)	.8
PROM	3 (6.3%)	7 (7.0%)	.8
Repeat cesarean delivery	31 (64.6%)	64 (64.0%)	.9
Operative time (minutes)	78.2±19 (55-120)	60.4±10 (45-90)	<.001
Hospital stay (days)	3.6±0.86	3.4±1.2	.21
Preoperative hemoglobin (g)	12.5±5.7	13.4±6.2	.35
Postoperative hemoglobin (g)	11.4±4.5	12.2±5.3	.38

Values are mean (SD) or number of patients and percentage. CPD: cephalopelvic disproportion; PROM: premature rupture of membranes

**Table 3:** Postoperative analgesic use and complications (group I: combined hernia and cesarean delivery; group II: cesarean delivery alone).

	Group I (n=48)	Group II (n=100)	*P* value
Analgesic ampule use (mean per week)	4.97 (1.2%)	4.59 (0.9%)	.027
Wound sepsis	2 (4.2%)	4 (4.0%)	.26
Seroma	1 (2.1%)	2 (2.0%)	.31
Wound disruption	0 (0.0%)	1 (1.0%)	.17

Values are mean (SD) or number of patients and percentage.

## DISCUSSION

Paraumbilical hernias are prone to incarceration and continue to enlarge if untreated, and thus they should be considered for repair at presentation.^8^ Our analysis of the potential benefit of combining cesarean delivery with hernia repair resulted in no increase in complications (notably infection) over cesarean delivery alone. Furthermore, all patients continued to be satisfied with the combined procedure up to 3 years later. The practical benefits were obvious: a 2-in-1 operation, with a single incision, single anesthesia, and single hospital stay, conferring valuable advantages for both patient and hospital in time, cost, and convenience, not to mention avoiding the separation of mother from newborn entailed by re-operation. In our opinion, the intraoperative difficulty of mesh fixation and to a lesser extent primary suture repair is the main problem, which always requires assistance to achieve good traction. Proponents of postpartum hernia repair may argue that the combined procedure increases the complication rate, because of blood loss and wound infection resulting from the longer operation time, and prolongs hospitalization. Our data refute this view. Hernia repair prolonged the average duration of cesarean delivery, but the time remained within the normal range reported for hernia repair in the literature.^9^ In all patients undergoing paraumbilical hernia repair, operation times remained below 120 minutes with a wound infection rate in our series of 4.2%, a little lower than the reported 6.2% within this time limit.^9^ Wound healing was delayed, with infection, in 6 patients. Hospitalization was not prolonged in patients undergoing the combined procedure. Disruption of the abdominal incision is a major source of morbidity after cesarean delivery. Failure of the abdominal skin incision to heal commonly occurs because of infection, abscess, hematoma, and seroma formation. Postoperative abdominal wound infection is a common cause of morbidity and has been reported to occur in 5% of patients.^10^,^11^ In our series seroma formation occurred in 3 patients (2%) and wound disruption occurred in 1 patient (0.68%). Forty-eight cases may appear few, but to our knowledge there is no equivalent series in the literature except for a series of 8 patients with inguinal as will as umbilical hernias.^5^ In these 8 cases, only 3 had umbilical hernia repaired during cesarean delivery and complication and recurrence rates were zero. Despite the high frequency of the umbilical hernia repair procedures, disappointingly high recurrence rates, up to 54% for simple suture repair, are reported.^12^ Large differences in hernia recurrence rates have been reported: between 10% to 30%, depending on surgical technique, length of follow-up, and method of recurrence assessment.^12^ Rates also increase with time, with most recurrences occurring early, in the first 3 months after mesh repair.^13^–^14^ In our study, the recurrence rate was 2.8% for the suture repair group and there was no recurrence in the mesh group, which is low if compared with the above reported rates.

Whether it is best to repair a pregnancy hernia during the pregnancy itself, at cesarean delivery or postpartum after involution of the uterus, remains a matter of dispute. Some authors recommend repair during pregnancy only in the case of strangulation and incarceration. Some have recommended repair of irreducible umbilical hernias before the enlarging uterus caused possible strangulation.^15^ Otherwise, if there are no complications during pregnancy, repair can be deferred until as soon as possible postpartum. One reason for deferral is that anesthesia and surgery during pregnancy could precipitate uterine irritability and induce premature labor, up to one week postoperatively.^15^ Other proposed reasons have been the extreme vascularization of the uterus, and the induction of collagen remodeling by relaxin during pregnancy, with the softened tissue predisposing to hernia recurrence.^16^

In conclusion, cesarean delivery combined with paraumbilical hernia repair avoids rehospitalization and appears safe, effective, and well accepted. It neither increased the complication rate nor prolonged the hospital stay, and in our sample, it was associated with acceptable longnterm recurrence (only one case). Confirmation of these results in a larger study, including other types of ventral hernia, would establish combined cesarean delivery and hernia repair as a recommendable procedure.
